# MiR-18a-3p improves cartilage matrix remodeling and inhibits inflammation in osteoarthritis by suppressing PDP1

**DOI:** 10.1186/s12576-022-00827-3

**Published:** 2022-02-11

**Authors:** Xiaoguang Feng, Jiajun Lu, Yixiong Wu, Haiyun Xu

**Affiliations:** Department of Orthopedics, Changzhou Cancer Hospital Affiliated to Soochow University, No.68 Honghe Road, Xinbei District, Changzhou, 213000 Jiangsu China

**Keywords:** OA, miR-18a-3p, PDP1, Chondrocyte, Inflammation

## Abstract

Osteoarthritis (OA) is a degenerative disease characterized by synovial inflammation. MiR-18a-3p was reported to be downregulated in knee anterior cruciate ligament of OA patients. In the present study, the specific functions and mechanism of miR-18a-3p in OA were explored. An in vitro model of OA was established using 10 ng/ml IL-1β to treat ATDC5 cells, and medial meniscus instability surgery was performed on Wistar rats to establish in vivo rat model of OA. RT-qPCR revealed that miR-18a-3p was downregulated in IL-1β-stimulated ATDC5 cells. MiR-18a-3p overexpression inhibited secretion of inflammatory cytokines and concentration of matrix metalloproteinases, as shown by ELISA and western blotting. The binding relation between miR-18a-3p and pyruvate dehydrogenase phosphatase catalytic subunit 1 (PDP1) was detected by luciferase reporter assays. MiR-18a-3p targeted PDP1 and negatively regulated PDP1 expression. Results of rescue assays revealed that PDP1 upregulation reserved the suppressive effect of miR-18a-3p overexpression on levels of inflammatory cytokines and matrix metalloproteinases in IL-1β-stimulated ATDC5 cells. H&E staining was used to observe pathological changes of synovial tissues in the knee joint of Wistar rats. Safranin O-fast green/hematoxylin was used to stain cartilage samples of knee joints. MiR-18a-3p overexpression suppressed OA progression in vivo. Overall, miR-18a-3p improves cartilage matrix remodeling and suppresses inflammation in OA by targeting PDP1.

**Supplementary Information:**

The online version contains supplementary material available at 10.1186/s12576-022-00827-3.

## Introduction

Osteoarthritis (OA) is an articular joint disease with high morbidity in the elderly people [1]. From 2005 to 2015, approximately 200 million of people were afflicted with OA, 2% of whom suffered from physical disability [2]. The etiological factors of OA include obesity, heredity, cardiovascular diseases, aging, genetic factors and joint injury [3]. OA is attributed to premature onset of OA after joint injury [4]. Clinically, the manifestation of OA includes narrowing of the joint space, emergence of osteophytes through osteoarthritic remodeling, cartilage erosion, and fibrillation [5]. In recent years, mounting medical expenditures used for OA treatment have brought a burden on OA patients and the whole society [6]. Therefore, there is an urgent need to look for an effective therapy of OA.

The main pathological changes of OA are articular cartilage degeneration, inflammation of synovitis, and secondary bone hyperplasia [7, 8]. Cartilage, a connective tissue with high specialization, is composed of chondrocytes and extracellular matrix [9]. Chondrocytes synthesize extracellular matrix to maintain the structural and functional integrity of the cartilage [10]. However, chondrocytes are difficult to maintain cartilage homeostasis in response to OA stimulation [11]. Chondrocytes convert to catabolic cells that secrete matrix-degrading enzymes, such as matrix metalloproteinases (MMPs), and cartilage degeneration was induced when catabolic-degrading effects overwhelm the anabolic-protective function in OA chondrocytes [10]. Chondrocyte catabolism can be stimulated by inflammatory cytokines including tumor necrosis factor α (TNF-α), interleukin 1β (IL-1β) and prostaglandin E2 (PGE2) [10]. TNF-α, for example, can induce the expression of proteases such as MMP1, MMP2, MMP3, MMP8, MMP9, MMP14, ADAMTS-4 and ADAMTS-5 [12]. Many previous studies also verified that inflammation in the synovitis is a key factor leading to OA progression [10, 13, 14].

MicroRNAs (miRNAs) are noncoding RNAs with 22–25 nucleotides in length [15]. The involvement of miRNAs in OA was reported in numerous studies [16]. For example, miR-485-3p regulates the Notch2/NF-κB pathway to inhibit apoptosis and promote chondrocyte proliferation in OA [17]. MiR-132 upregulation facilitates the proliferative ability and suppresses the apoptosis of chondrocytes in OA by modulating the PTEN/PI3K/AKT signaling pathway [18]. Overexpressed miR-144-3p reduces the numbers of IL-1β-positive cells in synovial tissue to ameliorate OA pathogenesis [19]. MiR-18a-3p was reported to be downregulated in knee anterior cruciate ligament of OA patients [20]. Additionally, miR-18a-3p targets HOXA1 to induce apoptosis of chondrocytes in OA [21]. Although miR-18a-3p can participate in OA pathogenesis, its effects on cartilage matrix remodeling and inflammation in OA are still unknown.

In this study, we explored the functional role of miR-18a-3p in cartilage matrix deposition and inflammatory response of chondrocytes in OA and investigated the underlying mechanism of miR-18a-3p. The study may help us understand the molecular mechanism underlying OA development and provide insight into effective therapies for OA patients.

## Material and methods

### Bioinformatic analysis

The potential target mRNAs of miR-18a-3p were predicted by miRDB (http://mirdb.org/) (screening condition: score rank ≤ 8), and the specific binding site between miR-18a-3p and PDP1 3′UTR was predicted by Targetscan (http://www.targetscan.org/).

### Adeno-associated virus (AAV) injection

To overexpress miR-18a-3p in joint tissues, the AAV serotype 2 containing miR-18a-3p mimics or miR-NC were constructed and packaged by Hanbio Company (Shanghai, China). After surgery, AAV-miR-18a-3p (1 × 10^9^ PFUs in a total volume of 5 μl) and the control AAV-miR-NC (5 μl) were intra-articular (IA)-injected into OA rats (*n*  = 8 rats) or sham-operated rats (*n*  = 8 rats).

### Establishment of OA animal model

A total of 32 Wistar rats (male, 8-week-old) were obtained from the Jackson Laboratory (USA) and were randomly divided into four groups: sham  +  AAV-miR-NC, sham  +  AAV-miR-18a-3p, OA  +  AAV-miR-NC and OA  +  AAV-miR-18a-3p. Each group has 8 rats. All rats were kept in a pathogen-free environment, and all operations were performed under isoflurane anesthesia, with all efforts made to minimize suffering. In OA group, rats were performed medial meniscus instability surgery on the right knee after anesthetization with isoflurane to induce joint instability and posttraumatic OA. Rats in sham group received similar surgical procedure without removing the ligament or meniscus. After surgery, AAV-miR-NC and AAV-miR-18a-3p were injected into OA rats or sham-operated rats. After 8 weeks, all rats were euthanized. Then, synovial tissues, cartilage tissues and blood samples were collected. All animal experiments were approved by the Animal Care and Use Committee of Changzhou Cancer Hospital Affiliated to Soochow University (Jiangsu, China) and performed in accordance with the committee’s guidelines.

### Cell culture and treatment

The chondrogenic cell line ATDC5 (Riken BioResource Center, Tsukuba, Japan) was incubated in DMEM/F12 (Gibco, Massachusetts, USA) supplemented with 10% FBS (Gibco) at 37 °C with 10% CO_2_. ATDC5 cells were treated with 10 ng/ml IL-1β (Roche Diagnostics, Germany) for 2 h and cells with DMSO (0.1%) treatment served as the control.

### Cell transfection

MiR-18a-3p mimics was used to overexpress miR-18a-3p and the negative control (NC) was named as miR-NC. Full-length sequence of pyruvate dehydrogenase phosphatase catalytic subunit 1 (PDP1) was subcloned into the pcDNA3.1 vector to elevate PDP1 expression with pcDNA3.1 as a control. All plasmids were constructed by Genepharma (Shanghai, China). Cell transfection was conducted using Lipofectamine 2000 (Invitrogen, USA) according to the manufacturer’s protocols, and the transfection efficiency was examined using reverse transcription quantitative polymerase chain reaction (RT-qPCR) after 48 h.

### Hematoxylin–eosin (H&E) staining

First, synovial tissues collected from sham-operated control rats and OA rats were fixed with paraformaldehyde, dehydrated, paraffin-embedded, and sliced into sections. After that, H&E was used to stain the tissues. A microscope was used to observe pathological changes of synovial tissues. The standard of pathological score was set as previous described [22]. Higher score represented greater joint injury severity.

### Safranin O/fast green staining

Knee joints of sham-operated control rats and OA rats were deparaffinized in xylene, hydrated with gradient ethanol, and stained with safranin-O/fast green. Scoring criteria was introduced in this study [23].

### Enzyme-linked immunosorbent assay (ELISA)

For in vitro experiments, ATDC5 cells (2 × 10^5^ cells/well) were pretreated with different vectors in 6-well plates for 2 h and then stimulated with IL-1β. For in vivo experiments, blood samples were collected from rats infected with AAV. Then, serum was obtained after blood samples were centrifuged at 6000×*g* for 15 min at 4 °C. The concentrations of inflammatory cytokines (IL-6, IL-8 and PGE2) in cells or serum were assessed using corresponding ELISA kits (Elabscience Biotechnology, Wuhan, China). Absorbance values were read at 450 nm in a microplate reader (BioTek synergy HT, Bedfordshire, UK).

### Western blotting

The concentration of total proteins obtained from ATDC5 cells and tissues of OA rats was determined by a BCA assay kit (Beyotime, Shanghai, China). Next, the proteins were loaded and separated by 8% SDS-PAGE. Then, the proteins were transferred onto PVDF membranes and blocked with 5% skim milk for 1 h. Afterwards, the membranes were incubated with primary antibodies including anti-MMP2 (ab92536; 1:1000), anti-MMP3 (ab52915; 1:1000), anti-MMP9 (ab76003; 1:1000), anti-PDP1 (ab198261; 1:200), and anti-β-actin (ab8226; 1:1000) at 4 °C overnight and then with secondary antibody horseradish peroxidase-labeled IgG (ab6721; 1:2000) at 4 °C for 2 h. Protein bands were visualized by BeyoECL Plus (Beyotime) and analyzed using Image Lab 3.0 (Invitrogen).

### Luciferase reporter assay

The wild-type (Wt) PDP1 3′UTR fragment containing binding site of miR-18a-3p was inserted into the pmirGLO vector (Promega, Madison, WI) to generate the luciferase reporter PDP1-Wt. PDP1-Mut was constructed by insertion of mutated PDP1 3′UTR into the pmirGLO vector. PDP1 3′UTR Wt/Mut were transfected into ATDC5 cells with miR-18a-3p mimic or miR-NC using Lipofectamine 2000 (Invitrogen).

### RT-qPCR

After transfection, the total RNA was extracted from ATDC5 cells and tissues of OA rats using TRIzol reagent (Invitrogen). Total RNA was reverse transcribed into cDNA utilizing a PrimeScript product RT reagent Kit (Takara, Kyoto, Japan). Then, cDNA was amplified by polymerase chain reaction (PCR). U6 was the internal reference of miR-18a-3p and GAPDH was the internal reference of mRNAs. The results were analyzed using the 2^−ΔΔCt^ method. The primer sequences are presented in Additional file 1: Table S1.

### Statistical analysis

The data are presented as the mean  ±  standard deviation and statistics were analyzed by GraphPad Prism software 6.0 (La Jolla, CA, USA). Student’s *t *test was utilized for difference comparison between two groups, and one-way analysis of variance followed by Tukey’s post hoc analysis were used for evaluation of differences among multiple groups. A value of *p*  < 0.05 was considered statistically significant.

## Results

### MiR-18a-3p overexpression improves cartilage matrix remodeling and inflammation in an in vitro model of OA

**Fig. 1:**
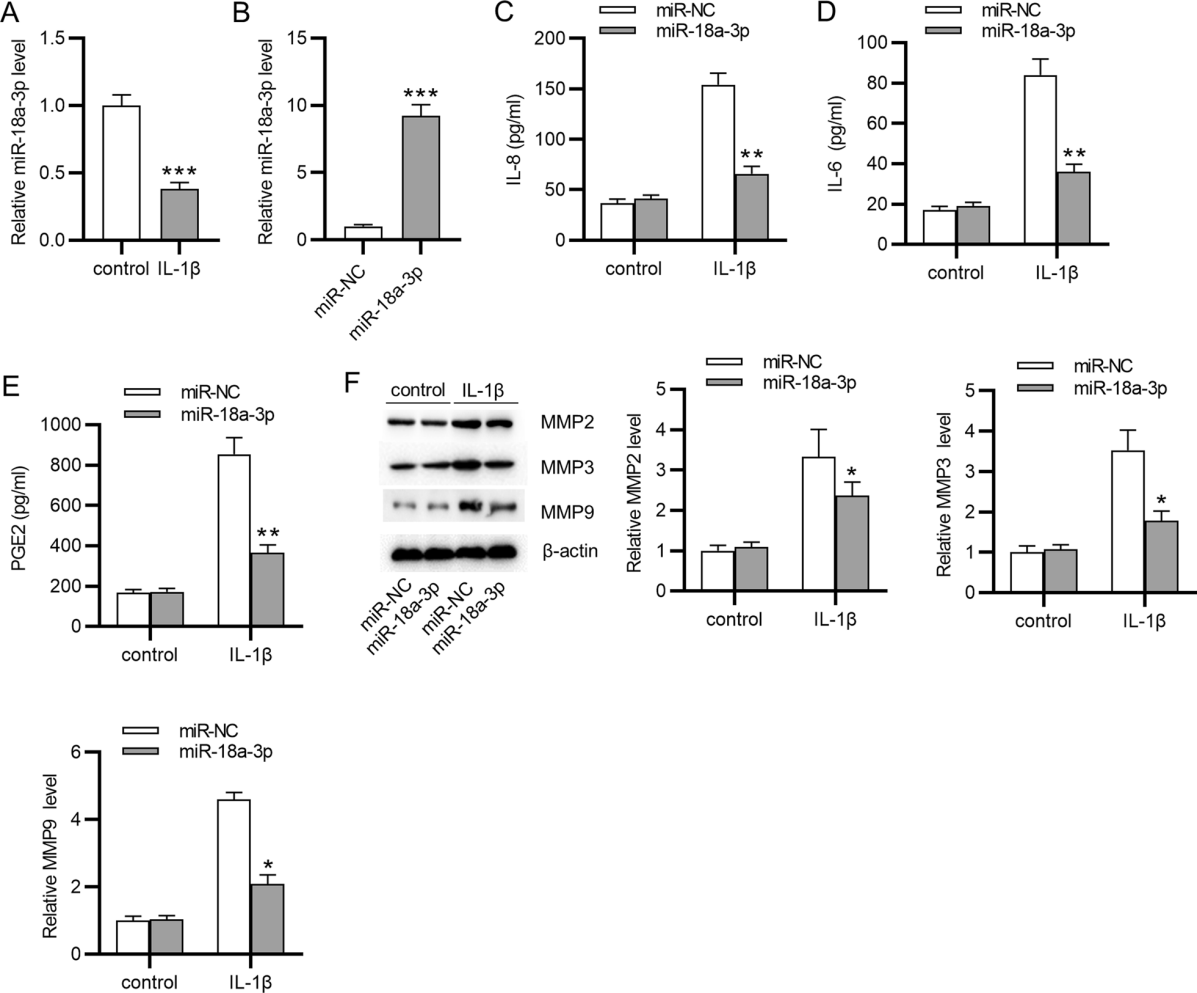
MiR-18a-3p overexpression improves cartilage matrix remodeling and inflammation in IL-1β-stimulated ATDC5 cells. **A** MiR-18a-3p expression in ATDC5 cells with or without IL-1β treatment was detected by RT-qPCR. **B** Transfection efficiency of miR-18a-3p mimics in IL-1β-stimulated ATDC5 cells was detected by RT-qPCR. After IL-1β treatment or miR-18a-3p overexpression, **C**–**E** the secretion of inflammatory cytokines IL-8, IL-6 and PGE2 in ATDC5 cells was examined by ELISA. **F** Protein levels of matrix metalloproteinases (MMP2, MMP3 and MMP9) were examined by western blotting. *p  < 0.05, **p  < 0.01, ***p  < 0.001

First, we used IL-1β to stimulate ATDC5 cells to construct an in vitro model of OA. After IL-1β treatment, we observed that miR-18a-3p expression in ATDC5 cells was downregulated (Fig. 1A). miR-18a-3p expression was increased in ATDC5 cells after transfection with miR-18a-3p mimic (Fig. 1B). Then, secretion of inflammatory cytokines including IL-8, IL-6 and PGE2 was examined by ELISA. The results showed that IL-1β increased the release of these inflammatory cytokines in ATDC5 cells, and miR-18a-3p overexpression reversed IL-1β-induced increase in cytokines (Fig. 1C–E). Meanwhile, the protein levels of matrix metalloproteinases (MMP2, MMP3, and MMP9) were increased in ATDC5 cells after IL-1β treatment compared with those in control groups and were decreased in IL-1β-stimulated ATDC5 cells silencing miR-18a-3p compared with those in cells transfected with miR-NC (Fig. 1F). All these data suggested that an in vitro model of OA was successfully established. In addition, miR-18a-3p is downregulated in IL-1β-stimulated ATDC5 cells, and miR-18a-3p overexpression suppress levels of matrix metalloproteinases and proinflammatory cytokines in the in vitro model of OA.

### MiR-18a-3p targets PDP1

**Fig. 2:**
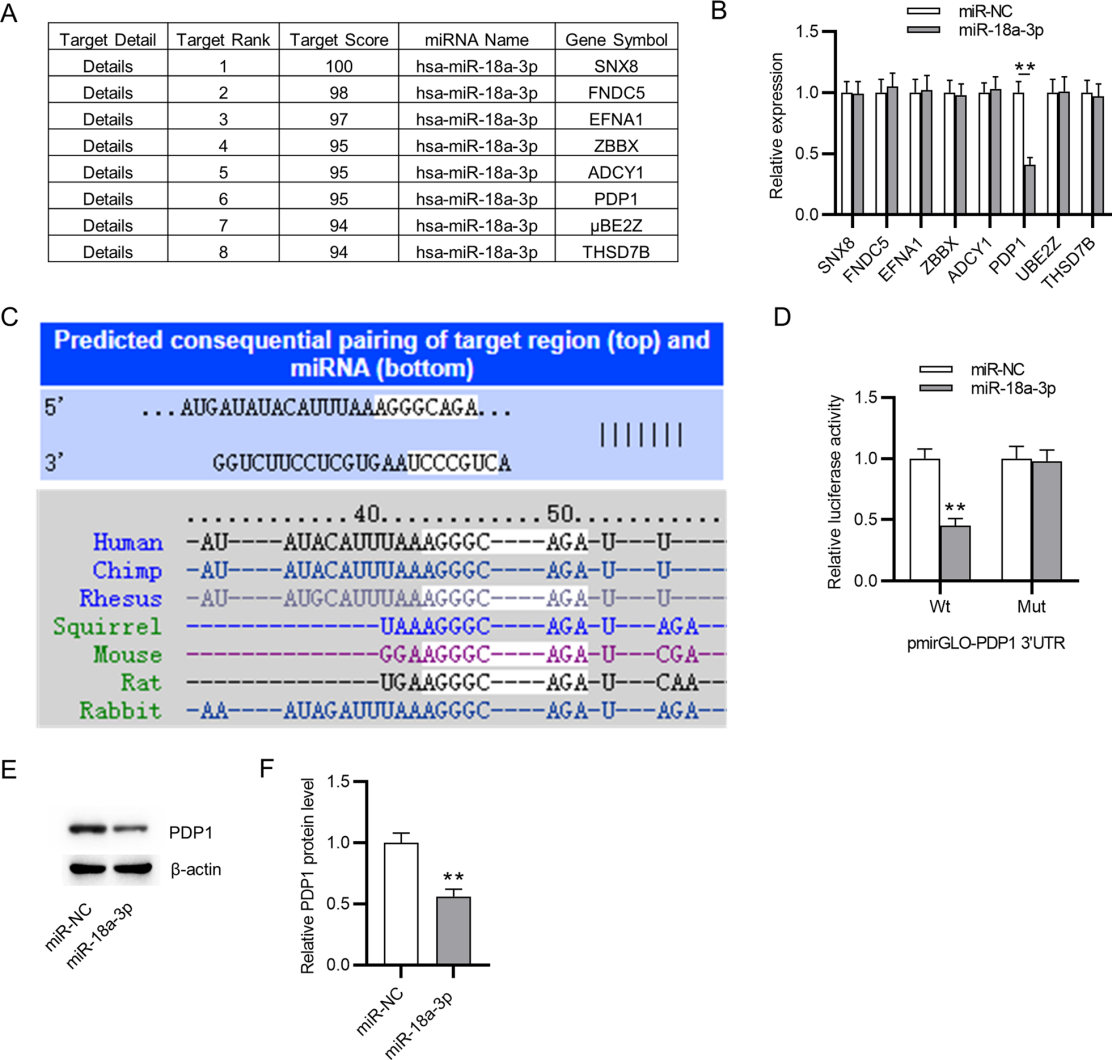
MiR-18a-3p targets PDP1. **A** Potential target genes (SNX8, FNDC5, EFNA1, ZBBX, ADCY1, PDP1, UBE2Z and THSD7B) of miR-18a-3p were predicted by miRDB. **B** The expression of potential target genes in ATDC5 cells transfected with miR-18a-3p mimics or miR-NC was detected by RT-qPCR. **C** The binding site of miR-18a-3p for PDP1 3′UTR was predicted by TargetScan. **D** Luciferase reporter assay was used to validate the binding relation between miR-18a-3p and PDP1 3′UTR in ATDC5 cells. **E**, **F** PDP1 protein level in ATDC5 cells after overexpressing miR-18a-3p was examined by western blotting. **p  < 0.01

To explore the mechanism of miR-18a-3p in IL-1β-stimulated ATDC5 cells, potential target genes of miR-18a-3p were predicted by miRDB and the first eight genes (SNX8, FNDC5, EFNA1, ZBBX, ADCY1, PDP1, UBE2Z and THSD7B) were identified (Fig. 2A). After overexpressing miR-18a-3p, only PDP1 was downregulated in ATDC5 cells among these candidates, as shown by RT-qPCR (Fig. 2B). The binding site of miR-18a-3p on PDP1 3′UTR was predicted by TargetScan, and the binding site is highly conserved among species (Fig. 2C). Luciferase reporter assay was used to validate the binding relation between miR-18a-3p and PDP1 3′UTR, which revealed that miR-18a-3p upregulation significantly decreased the luciferase activity of PDP1 3′UTR-Wt rather than that of PDP1 3′UTR-Mut (Fig. 2D). In addition, overexpressing miR-18a-3p contributed to the decrease in PDP1 protein level in ATDC5 cells (Fig. 2E, F). Therefore, it can be concluded that PDP1 is targeted by miR-18a-3p in ATDC5 cells.

### PDP1 upregulation reserves the inhibitory effect of miR-18a-3p overexpression on levels of inflammatory cytokines and matrix metalloproteinases in the in vitro model of OA

**Fig. 3:**
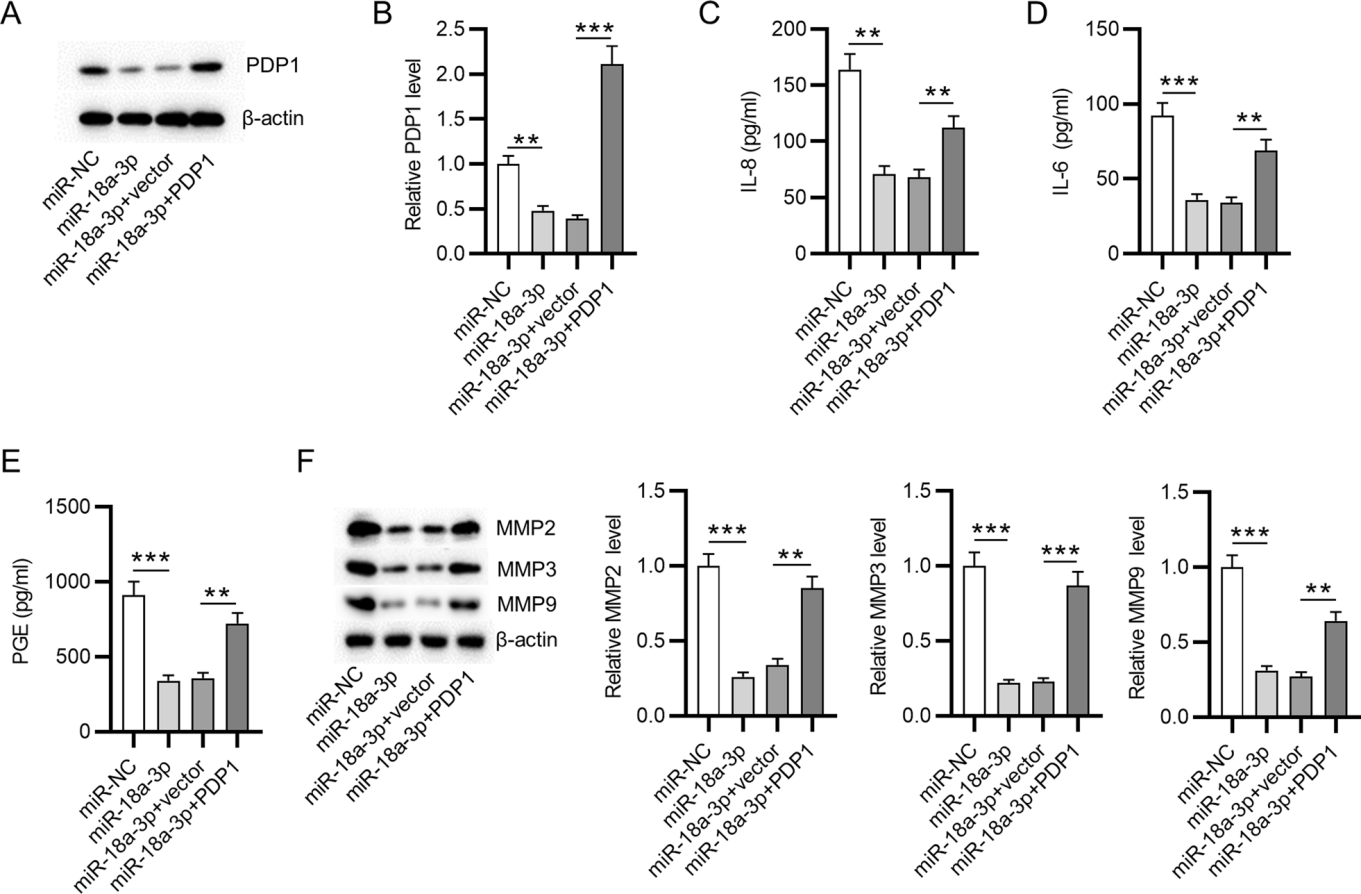
PDP1 upregulation reserves the inhibitory effect of miR-18a-3p overexpression on levels of inflammatory cytokines and matrix metalloproteinases in IL-1β-stimulated ATDC5 cells. **A**, **B** PDP1 protein level in IL-1β-stimulated ATDC5 cells after overexpressing miR-18a-3p or PDP1 was examined by western blotting. **C**–**E** The secretion of inflammatory cytokines IL-8, IL-6 and PGE2 in IL-1β-stimulated ATDC5 cells after overexpressing miR-18a-3p or PDP1 was examined by ELISA. **F** Protein levels of matrix metalloproteinases (MMP2, MMP3 and MMP9) in IL-1β-stimulated ATDC5 cells after overexpressing miR-18a-3p or PDP1 were examined by western blotting. **p  < 0.01, ***p  < 0.001

To identify the role of the miR-18a-3p/PDP1 regulatory axis in IL-1β-treated ATDC5 cells, rescue assays were performed. MiR-18a-3p overexpression downregulated PDP1 mRNA and protein levels in IL-1β-stimulated ATDC5 cells, while PDP1 upregulation reversed this trend (Fig. 3A, B). MiR-18a-3p-induced decrease in inflammatory cytokines (IL-8, IL-6 and PGE2) was reversed by PDP1 elevation in IL-1β-stimulated ATDC5 cells (Fig. 3C–E). Furthermore, miR-18a-3p upregulation led to reduction of matrix metalloproteinases (MMP2, MMP3 and MMP9) in IL-1β-stimulated ATDC5 cells, which was offset by PDP1 overexpression (Fig. 3F). Taken together, PDP1 upregulation reserves the suppressive effect of miR-18a-3p overexpression on levels of matrix metalloproteinases and proinflammatory cytokines in IL-1β-stimulated ATDC5 cells.

### MiR-18a-3p overexpression inhibits OA progression in vivo

**Fig. 4:**
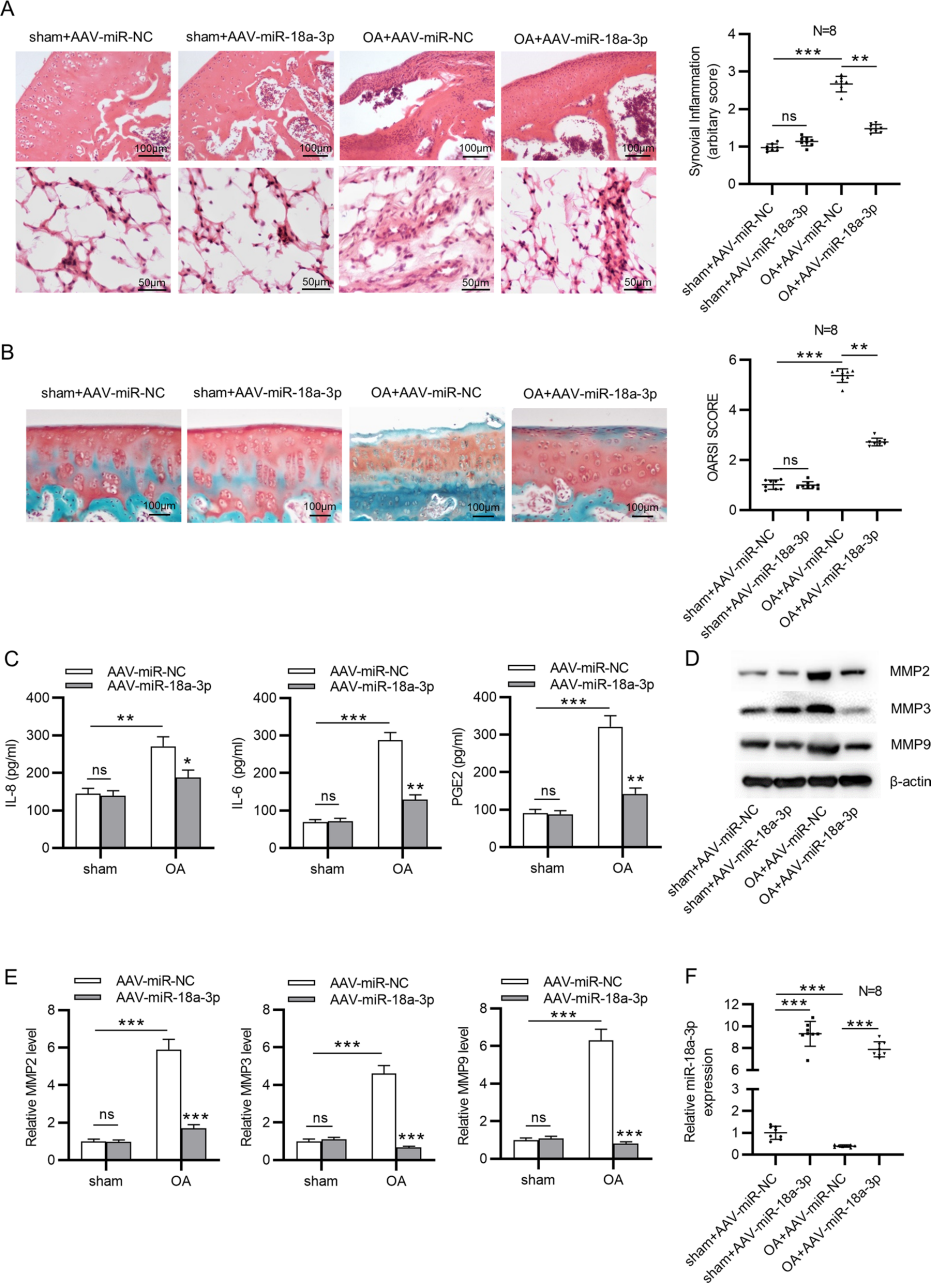
MiR-18a-3p overexpression inhibits OA progression in vivo. **A** H&E staining was used to observe pathological changes of synovial tissues in the knee joint of sham-operated control rats and OA rats treated with AAV-miR-NC or AAV-miR-18a-3p. **B** Safranin O-fast green/hematoxylin was used to stain histologic samples of knee joints of sham-operated control rats and OA rats treated with AAV-miR-NC or AAV-miR-18a-3p. **C** Serum levels of IL-8, IL-6 and PGE2 in sham-operated control rats and OA rats infected with AAV-miR-NC or AAV-miR-18a-3p were examined by ELISA. **D**, **E** Protein levels of matrix metalloproteinases (MMP2, MMP3 and MMP9) in sham-operated control rats and OA rats treated with AAV-miR-NC or AAV-miR-18a-3p were examined by western blotting **F** miR-18a-3p expression in cartilage tissues of rats of four groups was detected using RT-qPCR. *p  < 0.05, **p  <  0.01, ***p  < 0.001

To further validate the functions of miR-18a-3p in OA pathogenesis, in vivo experiments were also conducted. A total of 32 Wistar rats were randomly divided into four groups (*n*  = 8/group): sham  +  AAV-miR-NC; sham  +  AAV-miR-18a-3p; OA  +  AAV-miR-NC and OA  +  AAV-miR-18a-3p. According to the results from H&E staining, miR-18a-3p overexpression attenuated the pathological changes of synovial tissues in OA rats, including alleviated proliferation of synovial cells and reduced inflammatory cells (Fig. 4A). In addition, miR-18a-3p elevation significantly reduced cartilage degradation of OA rats according to results of Safranin O/Fast Green Staining (Fig. 4B). Moreover, serum levels of IL-8, IL-6 and PGE2 were higher in OA model rats than in sham-operated rats, and miR-18a-3p overexpression inhibited the secretion of inflammatory cytokines (Fig. 4C). The increase in matrix metalloproteinases (MMP2, MMP3 and MMP9) in OA rats was reversed after upregulating miR-18a-3p (Fig. 4D, E). Finally, miR-18a-3p expression in cartilage tissues was detected using RT-qPCR. After AAV-miR-18a-3p infection, miR-18a-3p was overexpressed in cartilage tissues of sham-operated rats (Fig. 4F). Compared with rats in the sham group, OA model rats exhibited decreased miR-18a-3p expression (Fig. 4F). Additionally, AAV-miR-18a-3p successfully upregulated miR-18a-3p in cartilage tissues of OA model rats (Fig. 4F). Overall, miR-18a-3p expression levels in vivo were consistent with results of miR-18a-3p expression detection in IL-1β-stimulated ATDC5 cells. Overall, miR-18a-3p elevation inhibits OA progression in vivo.

## Discussion

OA is a degenerative disease that poses a threat to health condition of humans [24]. IL-1β can induce articular chondrocytes to produce cytokines and chemokines, thus leading to inflammation [25]. IL-1β upregulates MMPs to damage articular cartilage structure and unbalance metabolism [26]. Thus, IL-1β overexpression is a hallmark of OA progression [27]. In this study, IL-1β was used to treat ATDC5 cells to stimulate the in vitro pathological environment of OA. After IL-1β stimulation, the secretion of inflammatory cytokines (IL-8, IL-6 and PGE2) and the concentration of matrix metalloproteinases (MMP2, MMP3 and MMP9) were increased in ATDC5 cells. The results suggested that an in vitro model of OA was successfully established.

**Fig. 5:**
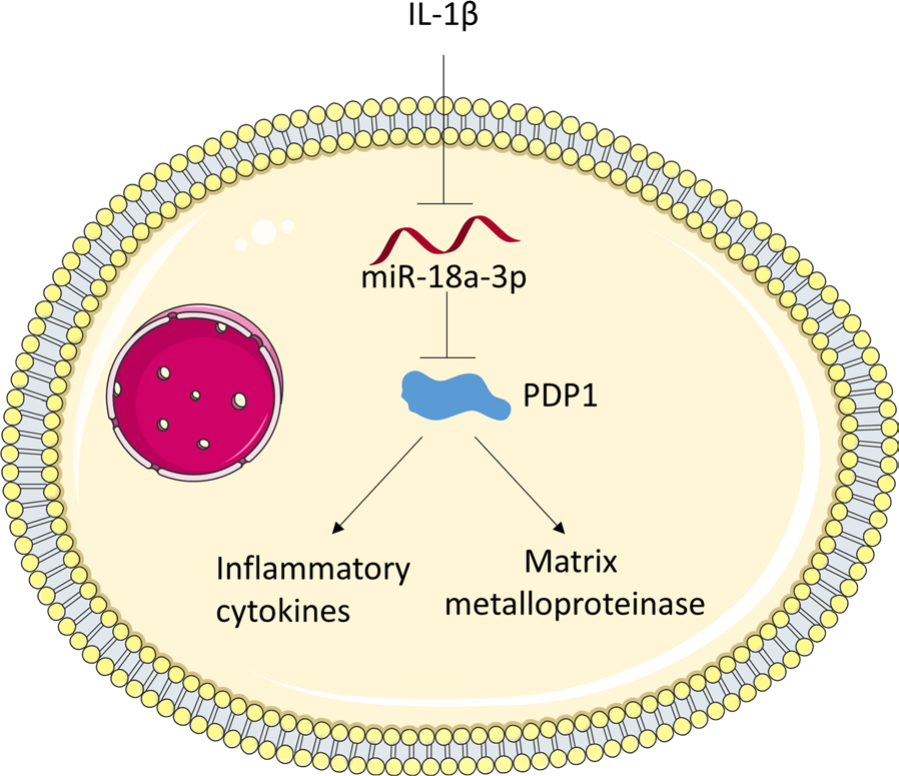
As shown in the diagram, IL-1β downregulates miR-18a-3p in ATDC5 cells. miR-18a-3p directly targets PDP1, and PDP1 promotes the concentration of inflammatory cytokines and matrix metalloproteinase. Thus, miR-18a-3p suppresses levels of inflammatory cytokines and matrix metalloproteinase by targeting PDP1 in IL-1β-stimulated ATDC5 cells

Accumulating studies have revealed that miRNAs are involved in OA progression [16, 28, 29]. In the current study, miR-18a-3p was downregulated in an in vitro model of OA, suggesting that miR-18a-3p participated in the pathogenesis of OA. MiR-18a-3p was reported to be downregulated in the knee anterior cruciate ligament of OA patients [20], which is consistent with our results. However, in another study, miR-18a-3p was reported to be upregulated in chondrocytes isolated from mouse and human cartilage tissues and directly target downstream gene homeobox A1 to facilitate chondrocyte apoptosis [21], which is opposite to our findings and the results of miRNA sequencing done by Bin et al. [20]. Furthermore, miR-18a, also termed miR-18a-5p, is derived from the same pri-miRNA as miR-18a-3p. MiR-18a-5p is overexpressed in primary Sjögren’s syndrome, a disease that can cause muscle and joint pain and muscle weakness [30]. MiR-18a-5p increased the expression of MMP1, inflammatory cytokines and chemoattractant proteins in rheumatoid arthritis synovial fibroblasts through NF-κB dependent manner after TNFα stimulation [31]. The previous study concluded that miR-18a-5p is an enhancer of TNFα-induced cartilage destruction and chronic inflammation in the joint [31]. These published articles revealed that miR-18a-3p and miR-18a-5p might play a different role in arthritis. Moreover, the functions of a miRNA might be affected by its upstream or downstream genes. In the study revealing the miR-18a-3p/HOXA1 axis in OA [21], HOAX1 inhibits chondrocyte apoptosis and act as a protective role in OA progression; in the study revealing the enhancer role of miR-18a-5p in TNFα-induced cartilage destruction and chronic inflammation [31], NF-κB is an inducer of miR-18a-5p and NF-κB activation is known to be involved in many chronic inflammatory diseases [32]. In the current study, the target gene PDP1 is identified to promote inflammation in many diseases [33–35]. Thus, we suspected that the different role of upstream or downstream genes may affect the functions of miR-18a-3p and miR-18a-5p in arthritis. Here, we also investigated the functions of miR-18a-3p in ATDC5 cells after IL-1β treatment. We found that IL-1β-stimulated the increase in concentration of inflammatory cytokines and matrix metalloproteinases was attenuated by miR-18a-3p upregulation in ATDC5 cells. More importantly, miR-18a-3p alleviated the pathological changes of OA rats in in vivo experiments. In conclusion, miR-18a-3p inhibits OA progression by improving cartilage matrix remodeling and suppressing inflammation (Fig. 5).

MiRNAs are gene regulators that directly bind to 3′UTR of their mRNAs to degrade transcripts or suppress protein translation [36, 37]. In the current study, PDP1 was identified as a target gene of miR-18a-3p and negatively regulated by miR-18a-3p. PDP1 is also called PDH, PDP, PDPC, PPM2A, PPM2C [38]. PDP1 overexpression leads to exhaustion of hematopoietic stem cells to increase the occurrence of myeloproliferative disorder [39]. Many studies revealed that induction of PDP1 expression is Notch-dependent, and PDP1 is involved in the activation of proinflammatory macrophages [33] and mouse hepatic macrophages [34]. PDP1 was also reported to function as an integral signaling target for proinflammatory stimuli (formyl-methionyl-leucyl-phenylalanine and leukotriene B4) and anti-inflammatory mediators (lipoxins) to regulate the activation of polymorphonuclear neutrophils in neutrophil proinflammatory responses [35]. Similarly, in this study, PDP1 upregulation reverses the inhibitory impact of miR-18a-3p upregulation on inflammatory cytokines in IL-1β-stimulated ATDC5 cells. The association between PDP1 and MMPs has not been clearly reported yet. Here, we discovered that overexpressing PDP1 rescued the suppressive effect on protein levels of MMP2, MMP3 and MMP9 in IL-1β-stimulated ATDC5 cells. All these results suggested that PDP1 promotes inflammatory response in OA development, and miR-18a-3p inhibits inflammation in OA by targeting PDP1.

In the published article, NF-κB is an inducer of miR-18a in TNFα-induced rheumatoid arthritis synovial fibroblasts [31]. Additionally, NF-κB activation is the principal pathway of Notch signaling pathways, and PDP1 is a target of the Notch pathway and is activated by Notch [33, 34]. The Notch pathway exerts a proinflammatory effect on OA [40]. Thus, we suspected that NF-κB or Notch signaling is associated with PDP1 expression in IL-1β-stimulated ATDC5 cells. However, whether NF-κB or Notch signaling is responsible for the downregulation of miR-18a-3p in IL-1β-stimulated ATDC5 cells and whether NF-κB or Notch signaling is involved in the miR-18a-3p/PDP1 axis still needs to be further investigated.

There are some limitations in our study. First, the present study lacked human biopsy examination to implicate the clinical relevance of our preclinical data. Second, the effects of PDP1 on chondrocytes and OA rats were not evaluated. Moreover, whether the miR-18a-3p/PDP1 axis is associated with the Notch pathway or NF-κB pathway in OA pathogenesis is still unknown. More relevant studies should be conducted in the future.

In conclusion, miR-18a-3p improves cartilage matrix remodeling and inflammation in OA by suppressing PDP1. Our study may offer a promising candidate for effective therapeutic method for OA progression.

### Supplementary Information

**Additional file 1: Table S1.** Sequences of primers used for RT-qPCR.

## Data Availability

The data underlying this article will be shared on reasonable request to the corresponding author.
