# Supplementary material for: MiR-18a-3p improves cartilage matrix remodeling and inhibits inflammation in osteoarthritis by suppressing PDP1
Source: J Physiol Sci. 2022 Feb 11;72:3. doi: 10.1186/s12576-022-00827-3 (PMC10717587; doi:10.1186/s12576-022-00827-3)
Supplement: Supplementary file 1 — Additional file 1: Table S1. Sequences of primers used for RT-qPCR. [file 12576_2022_827_MOESM1_ESM.docx]

| **Target** | **Sequences** |
| --- | --- |
|  | Forward, 5’-TCCAGACTGCCCTAAGTGC-3’ |
| miR-18a-3p | Reverse, 5’-CAGTGCGTGTCGTGGAGT-3’; |
|  | Forward, 5’-GGTGGTCAGGAACCAACAAC-3’ |
| PDP1 | Reverse, 5’-TCACAATCTCGCTGTTCTCG-3’ |
|  | Forward, 5’-GTGCTCGCTTCGGCAGC-3’ |
| U6 | Reverse, 5’- AAAAATATGGAACGCTTCACGAAT-3’ |
| GAPDH | Forward, 5’-CTGGCATTGCTCTCAATGAC-3’ |
|  | Reverse, 5’-CCGTATTCATTGTCATACCAGG-3’ |

**Supplementary table 1: The primer sequences**
